# Temperature Dependence of the Extrinsic Incubation Period of Orbiviruses in *Culicoides* Biting Midges

**DOI:** 10.1371/journal.pone.0027987

**Published:** 2011-11-18

**Authors:** Simon Carpenter, Anthony Wilson, James Barber, Eva Veronesi, Philip Mellor, Gert Venter, Simon Gubbins

**Affiliations:** 1 Vector-borne Disease Programme, Institute for Animal Health, Woking, Surrey, United Kingdom; 2 Entomology, ARC Onderstepoort Veterinary Institute, Hatfield, Pretoria, South Africa; 3 Department of Veterinary Tropical Diseases, University of Pretoria, Onderstepoort, South Africa; Duke-NUS Graduate Medical School, Singapore

## Abstract

**Background:**

The rate at which viruses replicate and disseminate in competent arthropod vectors is limited by the temperature of their environment, and this can be an important determinant of geographical and seasonal limits to their transmission by arthropods in temperate regions.

**Methodology/Principal Findings:**

Here, we present a novel statistical methodology for estimating the relationship between temperature and the extrinsic incubation period (EIP) and apply it to both published and novel data on virus replication for three internationally important orbiviruses (African horse sickness virus (AHSV), bluetongue virus (BTV) and epizootic haemorrhagic disease virus (EHDV)) in their *Culicoides* vectors. Our analyses show that there can be differences in vector competence for different orbiviruses in the same vector species and for the same orbivirus in different vector species. Both the rate of virus replication (approximately 0.017-0.021 per degree-day) and the minimum temperature required for replication (11-13°C), however, were generally consistent for different orbiviruses and across different *Culicoides* vector species. The estimates obtained in the present study suggest that previous publications have underestimated the replication rate and threshold temperature because the statistical methods they used included an implicit assumption that all negative vectors were infected.

**Conclusions/Significance:**

Robust estimates of the temperature dependence of arbovirus replication are essential for building accurate models of transmission and for informing policy decisions about seasonal relaxations to movement restrictions. The methodology developed in this study provides the required robustness and is superior to methods used previously. Importantly, the methods are generic and can readily be applied to other arbovirus-vector systems, as long as the assumptions described in the text are valid.

## Introduction

Arboviruses that utilise propagative biological transmission require a period of replication and dissemination within the arthropod vector (the *extrinsic incubation period*, EIP) [1]. As arthropods are poikilothermic, the temperature of their environment is a key factor affecting the rate at which an arbovirus is able to replicate to transmissible levels in a vector following ingestion. In the case of arboviruses which have recently emerged into new regions, such as bluetongue virus (BTV), a better understanding of the relationship between environmental temperature and virus replication is essential when trying to predict geographical and seasonal limits of transmission [2], [3], [4]. Furthermore, this information could inform policies currently used to limit the economic impact of an outbreak, such as the timing of animal movement restrictions, which are currently based on entomological activity [5].

Orbiviruses are vector-borne pathogens transmitted between vertebrate hosts by haematophagous arthropods. Three orbivirus species, all of which are transmitted by *Culicoides* biting midges, are of particular importance: BTV, African horse sickness (AHSV) and epizootic haemorrhagic diseases virus (EHDV). BTV causes a non-contagious, infectious disease called bluetongue (BT) in ruminants, particularly improved breeds of sheep. This disease has come to particular attention over the past decade following an unprecedented series of economically damaging outbreaks in Europe [6], [7], [8]. African horse sickness is a disease of equids caused by AHSV, which rarely causes clinical disease in donkeys or zebra, but can cause mortality of up to 90% in horses [9], [10]. Finally, epizootic haemorrhagic disease (caused by EHDV) often results in death in white-tailed deer (*Odocoileus virginianus*) and, less frequently, a bluetongue-like illness in cattle [11].

Several previous studies have examined the replication of orbiviruses in *Culicoides* incubated at controlled temperatures under laboratory conditions [12], [13], [14], [15], [16]. However, these studies relied on small numbers of insects, a problem compounded by the fact that a high proportion of individuals in any *Culicoides* population may be incapable of developing a fully disseminated infection [17], [18]. Furthermore, the statistical methods used to analyse the data were not ideal and did not fully reflect the experimental design. Finally, virus strains used in some previous studies were subjected to a relatively high degree of tissue passage, which may alter their ability to replicate in the insect vector [19], [20].

In this paper we develop novel statistical methods for analysing the temperature dependence of the EIP of arboviruses, which correctly reflect the experimental design and allow rigorous comparison of differences in replication rate amongst orbiviruses and vector species. These methods are applied to new experimental data in which a substantial number of *Culicoides sonorensis* (a confirmed vector species) were experimentally infected with a strain of BTV subjected to relatively few tissue passages. The methods are also applied to previously published data on orbivirus replication [14], [15] and the resulting parameter estimates are compared with those derived previously [15], [16]. We discuss limitations to previous methodologies used to characterise the EIP in *Culicoides*-orbivirus systems (in addition to those pointed out by [16]) and compare them with methods used for mosquito vectors. Finally, we discuss the application of modelling in the implementation of control measures against arboviral spread with reference to the recent BTV outbreak in northern Europe.

## Materials and Methods

### Laboratory determination of the EIP of BTV-9 (Kosovo) in *Culicoides sonorensis*

Groups of 2-3 day old *C. sonorensis* from the PIRB-s-3 strain [21] of the Pirbright colony [22] were fed on a blood-virus suspension containing 1 mL of heparinized sheep blood (TCS Biosciences Ltd, UK), and 1 mL of BTV supernatant. A BTV serotype 9 strain isolated in Kosovo (sample KOS2001/02 in the EU community reference laboratory collection; http://www.reoviridae.org/dsRNA_virus_proteins/ReoID/btv-9.htm) was used. This strain was selected for study as it represented the most northerly outbreak in Europe prior to the current BTV-8 epizootic and had been previously demonstrated to be capable of infecting UK populations of *Culicoides* [18]. The isolate had been subjected to a single passage through an 11 day old embryonated chicken egg and three passages through BHK-21 cells, and was used at a titre of 10^6.5^ tissue culture infectious dose 50 (TCID_50_)/mL when combined with blood in equal volume. Use of the chick embryo was conducted under Home Office project licence PPL 70/5793.

Adult midges were blood-fed using an artificial feeding apparatus [23] , with a stretched membrane of Parafilm® (Cole-Parmer, UK) used in place of the chick skin in the original method. *Culicoides* were allowed to feed for 30 min and then anaesthetised briefly with CO_2_ to remove and discard non-feeding females and males. Blood-fed females were then placed in netted, waxed pill-boxes (Watkins & Doncaster, UK) and stored in incubators at temperatures of 15, 20, 25 and 30°C. The incubating *C. sonorensis* females were provided with 5% sucrose via a cotton wool pad applied to the netting and changed daily. At 15, 20, 25 and 30°C a sample of 25 females was removed immediately after the blood meal and then every day until too few were left for further sampling (10–23 days depending on temperature). In a subsequent experiment carried in a methodologically identical fashion but using 12°C incubation, a sample of 25 females was removed immediately after the blood meal and then every five days for 40 days.

Following incubation, individual midges were placed in autoclaved 1.5 mL Eppendorf tubes containing 100 µL of chilled Glasgow minimal essential medium (MEM) with 0.6% antibiotics (2.0 µg/mL Fungizone, 1000 IU/mL penicillin, 50 mg/mL neomycin, and 1000 IU/mL polymyxin). They were then homogenized using sterilized, motor-driven, polypropylene pestles. Nine hundred µL of MEM was then added to each sample, and the tubes were centrifuged at 12,000 *g* for 5 min. One hundred µL of the resulting supernatant was then used to prepare a 1∶10 diluted sample using MEM in an additional Eppendorf tube for each sample. Virus titrations were carried out in tissue culture grade, 96-well microtitre plates containing a monolayer of BHK-21 cells and 100 µL of MEM supplemented with 2% tryptose phosphate broth (Invitrogen, United Kingdom) and antibiotics. Then, 100 µL of each sample and of 1∶10, 1∶100 and 1∶1000 dilutions of each sample were inoculated onto plates in four replicates, together with a positive control of the original virus used (at 10^−4^ to 10^−7^ dilution), and a negative control of diluent. Plates were sealed and incubated in an incubator at 37°C with microscopic observation for cytopathic effects at 3 and 5 days. Where bacterial contamination occurred, samples were passed through a 0.2 µL disposable filter (Sartorius UK) using a 5 mL syringe. Titres of positive samples were calculated using the method of Spearman and Kärber [24]. *Culicoides sonorensis* females containing ≥2.5 log_10_ TCID_50_ of BTV were considered to have replicated the virus to transmissible levels [25], [26].

### Previously published data on the EIP of orbiviruses

Further data were extracted from two papers: one on the replication of four orbiviruses (AHSV-4, BTV-10, BTV-16 and EHDV-1) in colony *C. sonorensis* [15]; and one on the replication of BTV-1 in field-collected *C. bolitinos* and *C. imicola* in southern Africa [14]. The latter were re-analysed from original datasets and times to transmissible infections interpreted from the proportion of *Culicoides* possessing ≥2.5 log_10_ TCID_50_ of BTV/individual and the number and titre of infected midges during the previous time period.

Data from two other papers were not analysed. The first presented an investigation of the temperature dependence of the EIP of BTV-11 in colony *C. sonorensis* AK strain [12], but completion of the EIP was judged by ELISA absorbance values (correlated originally to plaque assay quantification) [27] rather than virus titres, making results difficult to compare. The second collated data on the EIP derived from a range of sources (see references in [13]) , but most investigated virus replication at a single temperature using a variety of different viruses and methods of virus detection. All the data used in the present analysis are summarised in Table 1.

**Table 1:** Summary of datasets on the EIP of orbiviruses and vector species used in this study.

orbivirus*	vector species	source	temperatures (°C)	N†	reference
AHSV-4	*C. sonorensis*	colony	15, 20, 25, 30	10	[15]
BTV-1	*C. bolitinos*	field	10, 15, 18, 23.5, 25, 30	varies	[14]
BTV-1	*C. imicola*	field	10, 15, 18, 23.5, 25, 30	varies	[14]
BTV-9	*C. sonorensis*	colony	12, 15, 20, 25, 30	25	new data; see text
BTV-10	*C. sonorensis*	colony	15, 20, 25, 30	10	[15]
BTV-16	*C. sonorensis*	colony	15, 20, 25, 30	10	[15]
EHDV-1	*C. sonorensis*	colony	15, 20, 25, 30	10	[15]

*AHSV - African horse sickness virus; BTV - bluetongue virus; EHDV - epizootic haemorrhagic disease virus; number indicates serotype.

† number of midges sampled at each time point.

### Modelling the relationship between EIP and temperature

The EIP was assumed to follow a gamma distribution with probability density function given by,
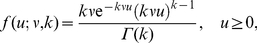
(1)where ν is the reciprocal of the mean EIP and *k* is the scale parameter (so that 1/ν and 1/*k*ν^2^ are the mean and variance for the distribution, respectively). The temperature dependence of the EIP is reflected in the reciprocal of the mean (i.e. ν) which is given by,
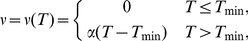
(2)where *T*_min_ is the threshold temperature for virus replication and α reflects the rate of replication above the threshold (with 1/α giving the number of degree-days above the threshold temperature required for a vector to complete the EIP).

In the laboratory experiments each midge tested can be in one of three states: (1) infected and has completed the EIP; (2) infected, but has yet to complete the EIP; or (3) uninfected. The probabilities that a midge tested *t* days post infection when infected at age *a* and reared at temperature *T* is in each of these states can be written as,
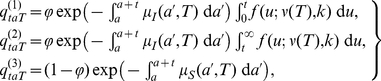
(3)where ϕ is the probability of becoming infected, *f* is the probability density function for the gamma-distributed EIP (see equation (1)) and µ*_I_*(*a*,*T*) and µ*_S_*(*a*,*T*) are the age- and temperature-dependent mortality rates for infected (*I*) and uninfected (*S*) vectors, respectively. The probability that a midge tested *t* days after infection when reared at temperature *T* has completed the EIP is given by,
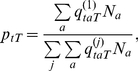
(4)where *N_a_* is the number of *Culicoides* of age *a* at the time of infection and the 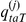 s are defined in equation (3). Assuming that (i) all *Culicoides* are the same age at infection; and (ii) there is no differential mortality between infected and uninfected vectors (i.e. µ*_I_*(*a*,*T*) =  µ*_S_*(*a*,*T*)), this probability, (4), simplifies to,
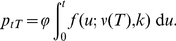
(5)


We note that the same expression would also be obtained if the vector mortality rates are independent of age (and there is no differential mortality).

### Parameter estimation

Model parameters were estimated in a Bayesian framework, which has two components: a likelihood function and a joint prior distribution for the parameters. The number of midges with a fully disseminated infection is drawn from a binomial distribution with the number of midges sampled and the probability that a sampled midge has completed the EIP (given by equation (5)), as parameters. Hence, up to a constant of proportionality, the likelihood for the data is given by,
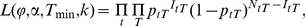
(6)where *I_tT_* is the number of midges with a fully disseminated infection and *N_tT_* is the number of midges sampled on day *t* after being reared at temperature *T*. Non-informative priors were used for the parameters: Uniform(0,1) for ϕ; diffuse Normal for α and *T*_min_; and diffuse exponential for *k*. The priors were assumed to be independent.

The joint posterior density for the parameters is proportional to,
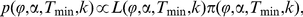
(7)where *L* is the likelihood, (6), and π(ϕ,α,*T*_min_,*k*) is the joint prior distribution for the parameters. Samples from the joint posterior distribution were generated using Markov chain-Monte Carlo (MCMC) methods, more specifically, a random-walk Metropolis algorithm [28]. For each data-set five chains of 50,000 iterations were run (with the preceding 10,000 iterations discarded to allow for burn-in of the chain) and were subsequently thinned (by selecting every fiftieth iteration) to reduce correlation amongst the samples. Convergence of the chains was monitored using various measures implemented in the “Convergence Diagnostics and Output Analysis” (CODA) package [29] in R [30]. Model fit was assessed by posterior predictive χ^2^ tests [31].

## Results

### Laboratory determination of EIP for BTV-9 (Kosovo) in *C. sonorensis*

**Figure 1:**
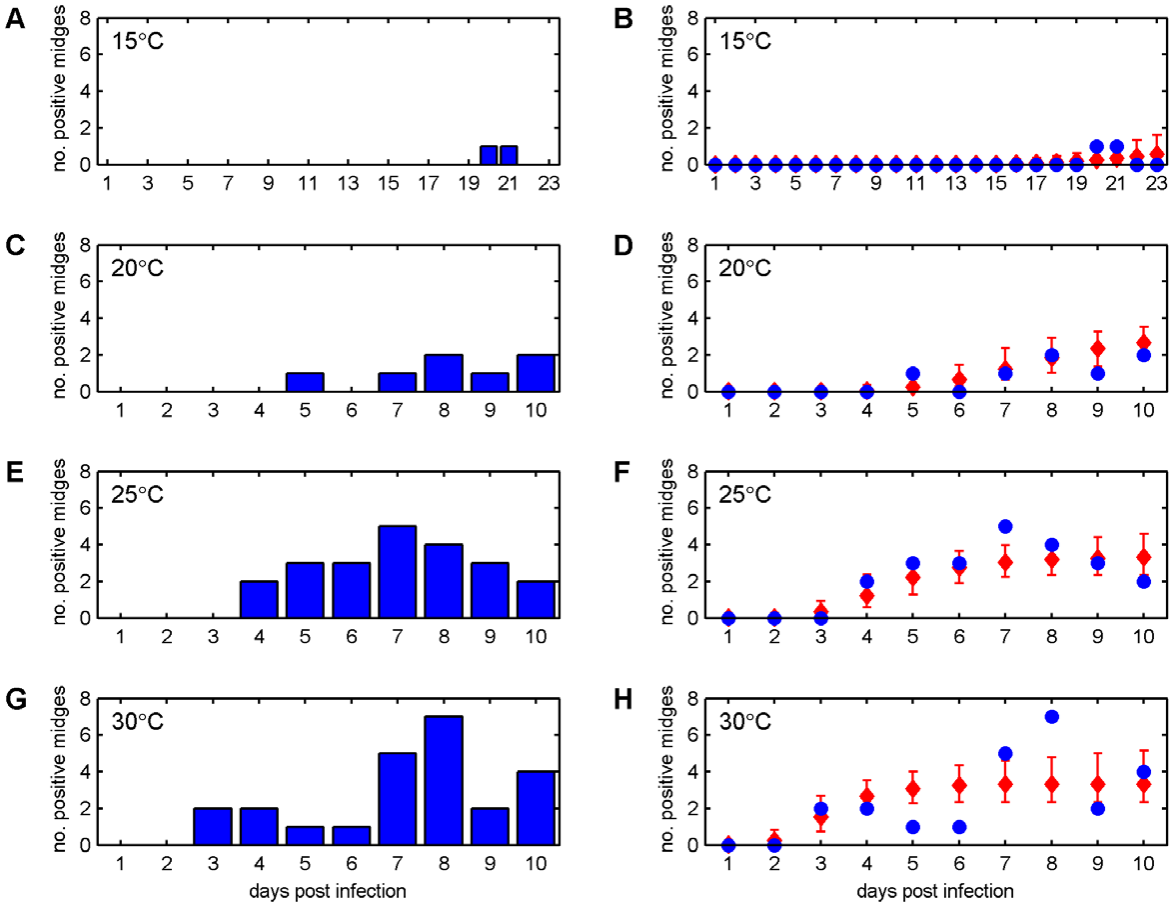
Observed and expected number of *Culicoides sonorensis* with a fully disseminated BTV-9 infection when reared at different temperatures. (A,C,E,G) Number (out of 25) of *C. sonorensis* with a fully disseminated infection. (B,D,F,H) Observed (blue circles) and expected (posterior median (red diamonds) and 95% credible interval (error bars)) number of *C. sonorensis* with a fully disseminated infection. Results are shown for midges reared at (A,B) 15°C, (C,D) 20°C, (E,F) 25°C, or (G,H) 30°C; those for midges reared at 12°C (no midges with a fully disseminated infection) are not shown.

The time to first transmissible infection of *C. sonorensis* by the strain of BTV used was three days at 30°C, four days at 25°C, five days at 20°C, and 20 days at 15°C (Figure 1A, C, E, G). No transmissible infections were recorded up to 40 days of testing at 12°C.

### Temperature-dependent model of the EIP

**Figure 2:**
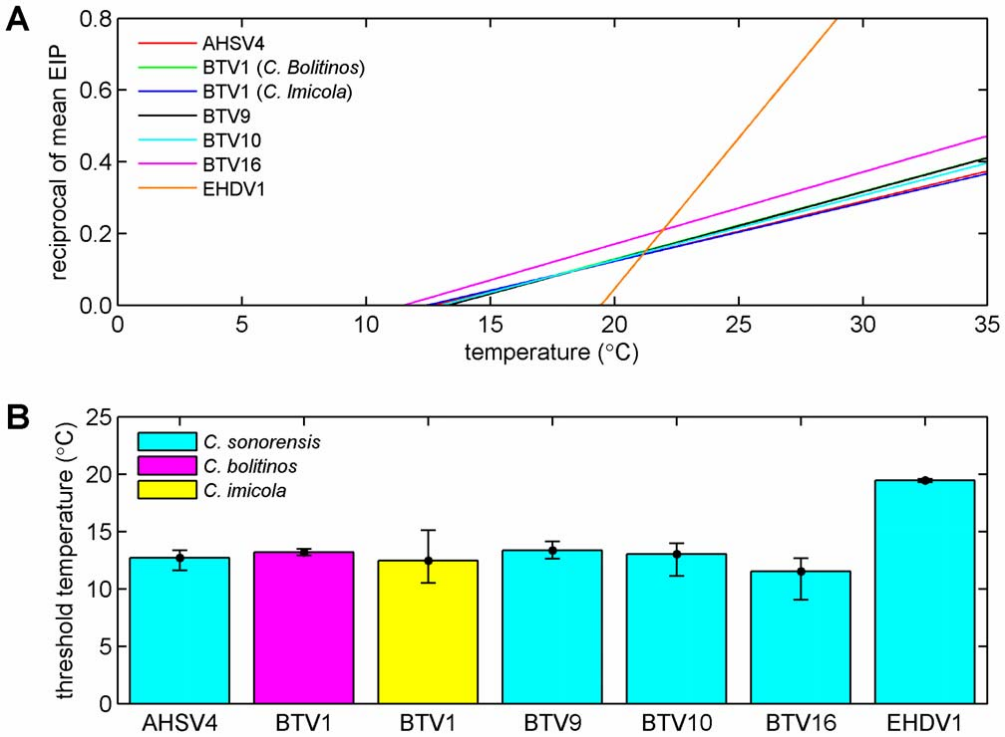
Temperature dependence of the extrinsic incubation period for six orbiviruses. (A) Temperature dependence of the reciprocal of the mean EIP (equation (2) using posterior median estimates for α and *T*_min_). Virus (and serotype) is indicated by the line colour (see legend). (B) Posterior median (bars) and 95% credible limits (error bars) for the threshold temperature for virus replication. Vector species is indicated by the bar colour (see legend).

The probability of infection (ϕ) ranged from 0.04 (BTV-1 in *C. imicola*) to 0.91 (EHDV-1 in *C. sonorensis*) (Table 2). The virus replication rate (α) was similar for all viruses and vectors except EHDV-1, for which the estimate was much higher (Figure 2A; Table 2). The threshold temperature (*T*_min_) was between 11.4°C and 13.3°C for the BTV and AHSV isolates, but was markedly higher (19.5°C) for EHDV-1 (Figure 2B; Table 2). Finally, estimates for the scale parameter (*k*) were often very high (Table 2), but were significantly (*P*<0.05) different from one for all orbiviruses in the study.

**Table 2:** Posterior mean, median and 95% credible limits (CL) for parameters in the temperature-dependent model of the extrinsic incubation period for different orbiviruses and vector species.

orbivirus	vector species	probability of infection (ϕ)				virus replication rate (α)			
		mean	median	95% CL		mean	median	95% CL	
				lower	upper			lower	upper
AHSV-4	*C. sonorensis*	0.52	0.52	0.45	0.59	0.017	0.017	0.014	0.020
BTV-1	*C. bolitinos*	0.61	0.61	0.58	0.64	0.019	0.019	0.018	0.020
BTV-1	*C. imicola*	0.04	0.04	0.03	0.07	0.016	0.016	0.010	0.023
BTV-9	*C. sonorensis*	0.14	0.13	0.09	0.20	0.019	0.019	0.013	0.026
BTV-10	*C. sonorensis*	0.12	0.12	0.08	0.18	0.018	0.018	0.010	0.029
BTV-16	*C. sonorensis*	0.16	0.15	0.12	0.20	0.021	0.020	0.016	0.028
EHDV-1	*C. sonorensis*	0.92	0.92	0.88	0.96	0.084	0.084	0.068	0.105

		threshold temperature (*T*_min_)				scale parameter (*k*)			
		mean	median	95% CL		mean	median	95% CL	
				lower	upper			lower	upper
AHSV-4	*C. sonorensis*	12.6	12.7	11.6	13.3	16.7	14.4	6.8	37.7
BTV-1	*C. bolitinos*	13.2	13.2	12.9	13.5	7.8	7.7	5.9	10.1
BTV-1	*C. imicola*	12.7	12.5	10.6	15.5	90.9	66.5	2.7	330.3
BTV-9	*C. sonorensis*	13.3	13.3	12.7	14.1	19.5	14.3	3.5	64.5
BTV-10	*C. sonorensis*	12.9	13.0	11.0	14.0	68.9	46.4	5.0	256.5
BTV-16	*C. sonorensis*	11.4	11.6	9.3	12.6	146.8	116.2	18.9	433.5
EHDV-1	*C. sonorensis*	19.5	19.5	19.3	19.6	6.9	6.6	3.4	12.2

Formal comparison of the parameter estimates for the different orbiviruses/vectors (by sampling from the joint posterior distributions) showed that those for the probability of infection (ϕ) were similar (*P*>0.05) for all the BTV isolates (9, 10 and 16) tested in *C. sonorensis*, but were significantly (*P*<0.01) different for the other orbiviruses/vector species tested. The virus replication rate (α) for EHDV-1 in *C. sonorensis* was significantly (*P*<0.001) higher than for the other orbiviruses/vector species (which did not differ from one another; *P*>0.05). Finally, the threshold temperature (*T*_min_) was significantly (*P*<0.001) higher for EHDV-1 in *C. sonorensis* compared with the other orbiviruses/vector species tested and for BTV-9 in *C. sonorensis* and BTV-1 in *C. bolitinos* compared with BTV-16 in *C. sonorensis* (*P*<0.01).

Posterior predictive checking indicated that the model provided an adequate fit to the data for all orbiviruses, except BTV-1 in *C. bolitinos*. A comparison of the observed and expected values for BTV-9 is shown in Figure 1B, D, F, H; those for the previously published data are shown in supporting information (Figure S1 for data from [15]; Figure S2 for data from [14]).

## Discussion

The results of our analyses highlight differences in vector competence for different orbivirus strains and vector species (Table 2). However, the rate of virus replication (approximately 0.017-0.021 per degree-day) and temperature (11-13°C) required for replication were broadly consistent across different orbivirus strains and vector species, with the exception of EHDV-1 (Figure 2; Table 2). The original data produced for EHDV-1 were extremely variable across the temperature range tested [15], however, and require confirmation in future studies.

Comparison of the estimates for the virus replication rate (α) and threshold temperature (*T*_min_) derived in the present study (Table 2) with those derived previously for AHSV-4, BTV-10, BTV-16 and EHDV-1 [15] indicate that the earlier methods substantially underestimate both α and *T*_min_ (Table S1). Moreover, using the estimates for α and *T*_min_ obtained in [15] (with ϕ and *k* estimated independently by maximum likelihood methods using equation (6)) shows a poor fit of the model to data for all four viruses (χ^2^ goodness-of-fit tests: *P*<0.05). In particular, there is a tendency to overestimate the number of positive midges at lower temperatures and underestimate the number of positive midges at higher temperatures when using the earlier parameters (Figure S1).

To explore the reasons for this inaccuracy, we simulated replicated data-sets for each of the four orbiviruses using the model given by equations (1)–(3) and the parameter estimates in Table 2. Applying the methods used in [15] to the simulated data produced similar estimates to those obtained by [15] from the experimental data. By contrast, if the methods used by [15] were applied to the simulated data, but only those for infected midges (which have or have not completed the EIP), they recover the parameter estimates obtained in the present study. This indicates that the earlier methods are limited because they fail to distinguish between infected vectors which have yet to complete the EIP and uninfected vectors.

Two assumptions were made when analysing the data: (i) there is no differential mortality between infected and uninfected vectors; and (ii) there is no age-dependent mortality (or, equivalently, all midges were the same age when infected). Differential mortality has not been studied for *Culicoides* spp. infected with orbiviruses, though there is evidence for increased mortality in mosquito vectors infected with a number of arboviruses (for example, chikungunya virus in *Aedes albopictus* [32]; eastern equine encephalomyelitis virus in *Culiseta melanura* [33]; or Rift Valley Fever virus in *Culex pipiens* [34]). Analysis of the modelling approach, (3)–(4), shows that estimates for the EIP parameters (α, *T*_min_ and *k*) will be unaffected if there is differential mortality, but the probability of infection (ϕ) will be underestimated. Regarding the second assumption (reviewed recently by [35]), there is some evidence for age-dependent mortality in *C. sonorensis*, but this appears to vary with geographic origin [36]. For the analyses of EIP data obtained from colony-derived *Culicoides* (Table 1), age-dependent mortality is unlikely to have an impact on the parameter estimates, because the midges used in the experiments had all emerged two to three days prior to infection and so were similar ages at infection. This could, however, be an issue for the data obtained from experiments using field-caught vectors (Table 1), where this is unlikely to the case, and may help account for the relatively poor fit of the model to data on the EIP of BTV-1 in *C. bolitinos*.

By constraining the scale parameter (*k*) to be an integer, it is straight-forward to implement the EIP as series of exponential distributions [37], which can be readily incorporated in orbivirus transmission models [2], [38]. In this case, the appropriate choice for the scale parameter is the nearest integer to the value given in Table 2, while the other parameters remain unchanged. Importantly, our results show that the scale parameter (*k*) differs significantly from one and, hence, the commonly-made assumption of an exponential distribution for the EIP [3], [4] is not appropriate for orbiviruses, meaning that other approaches to modelling their EIP should be used [2].

Experimental techniques for the laboratory investigation of the EIP of orbiviruses in *Culicoides*, and the competence of *Culicoides* for orbiviruses, have improved in recent years due the development of rapid detection methodologies (e.g. [39]) which facilitate high-throughput screening of the large numbers of uninfected *Culicoides* generated by vector competence or EIP studies [40], [41]. Studies have also shifted from using a hamster-derived cell line for detection purposes (BHK-21) that is prone to variation in sensitivity across lines, orbivirus strains and users, towards the *C. sonorensis*-derived KC cell line which has greater epidemiological relevance to orbivirus studies [27], [42]. However, methods for the analysis of the resulting data have not significantly improved during the same period. The method described here represents a substantial improvement upon previous studies, correctly reflecting the experimental design and allowing the rigorous comparison of differences in replication rate amongst orbiviruses and vector species.

Experimental limitations remain in the laboratory determination of the EIP of orbiviruses in *Culicoides* (see also [16]). Chief among these is the lack of a rapid technique to directly determine the degree of orbivirus dissemination within infected *Culicoides* and, hence, provide a realistic view of the probability of transmission of virus to the host [43]. In mosquitoes, salivary glands can be excised relatively straightforwardly and tested for virus [44], or potentially infected individuals can be stimulated to produce saliva which can subsequently be screened [45]. These techniques have been trialled for *Culicoides* [46] but the small size of the subjects makes processing the large numbers of individuals required for both EIP and vector competence studies prohibitively laborious. Instead, most recent studies have inferred transmissibility indirectly using the relationship between the whole-body titre of infected *C. sonorensis* and the presence of virus in the saliva of individuals (as estimated by [26]), or in blood from meals taken by *C. sonorensis* through a membrane based system [25].

The only study to directly trace dissemination in *C. sonorensis* over time, using an immunohistochemistry assay, found that in competent individuals held at 24±1°C virus disseminated rapidly from through the midgut and was present in the salivary glands by day five post-infection [26]. This contrasted with a previous study that hypothesised from profiles of virus replication recorded in pools of *C. sonorensis* held at 23°C that the period of replication and dissemination within the haemocoel, following entry through the midgut, was substantial and lasted up to 6 days [47]. Hence, while the presence of virus in saliva of *Culicoides* has been inferred for the current study as representing transmissible infections it is currently unclear what degree of replication in secondary organs is required to allow onwards transmission to the host. Further studies taking into account additional factors that may enhance transmission efficacy [48] and utilising appropriate live hosts, have the potential to clarify this relationship, particularly when combined with advances in technologies for visualising virus dissemination within the insect vector.

A second experimental limitation to current studies is that detailed investigations require the use of large numbers of colony-bred *Culicoides*. This has led to reliance upon the use of *C. sonorensis* lines due to the difficulty of colonising alternative African or European vector species. The successful production of blood feeding methods for field populations of *C. imicola* in Southern Africa has raised the potential of laboratory colonisation for this species [49]. Research on methodologies to produce sustainable colonies of those northern European species that have been implicated in BTV transmission have, however, been limited to date and require further examination.

Despite these qualifications, the results presented here remain the most robust to date for the orbivirus-*Culicoides* system. In terms of practical application, the results presented here could be used to inform the timing of livestock movement restrictions which are used to control the spread of BTV and other orbiviruses. Current EU legislation already permits the movement of unvaccinated animals during periods of minimal *Culicoides* activity, as measured using light trap networks (see [50] for a review). The earliest date on which it is possible for transmission from vectors to hosts to resume could be estimated from temperature-EIP relationships such as the ones described in the present study using thermal time accumulation models [51] similar to those used already to forecast crop growth [52] and the development of insect pests [53], provided the temperature of the environment in which the vectors rest after feeding is known. Such an approach has already been suggested for West Nile virus in the United States [54]. A strategy based upon a combined dataset of seasonal activity of *Culicoides* and a temperature-derived “transmission-free period” could potentially allow safe animal movements for longer periods and with more warning of revocation. This could lead to substantial economic benefits for the livestock industry, but is dependent upon accurate quantitative understanding of the relationship between temperature and EIP, emphasising the need for such relationships to be examined in detail using the listed recommendations to improve both the accuracy of laboratory studies and subsequent analyses.

## Supporting Information

Figure S1 Observed and expected number of *Culicoides sonorensis* with a fully disseminated infection when reared at different temperatures. Each figure shows the observed (blue circles) and expected (posterior median (red diamonds) and 95% credible interval (error bars)) number of positive *C. sonorensis* infected with different orbiviruses: African horse sickness virus (AHSV); bluetongue virus (BTV); and epizootic haemorrhagic disease virus (EHDV) (the number indicates serotype). The data were extracted from [15]. The cyan squares show the expected number of positive midges using the estimates for α and *T*_min_ obtained by [15] with ϕ and *k* estimated independently by maximum likelihood methods.
(TIF)

Figure S2 Observed and expected number of *Culicoides bolitinos* or *Culicoides imicola* with a fully disseminated infection when reared at different temperatures. Each figure shows the observed (blue circles) and expected (posterior median and 95% credible interval: red diamonds and error bars) number of positive *C. bolitinos* or *C. imicola* infected with bluetongue virus serotype 1. The data were extracted from [14].
(TIF)

Table S1 Previous estimates for the temperature dependence of the extrinsic incubation period of different orbiviruses in *Culicoides sonorensis*.
(DOCX)
