# Supplementary figures and images for: Temperature Dependence of the Extrinsic Incubation Period of Orbiviruses in Culicoides Biting Midges
Source: PLoS One. 2011 Nov 18;6(11):e27987. doi: 10.1371/journal.pone.0027987 (PMC3220716; doi:10.1371/journal.pone.0027987)

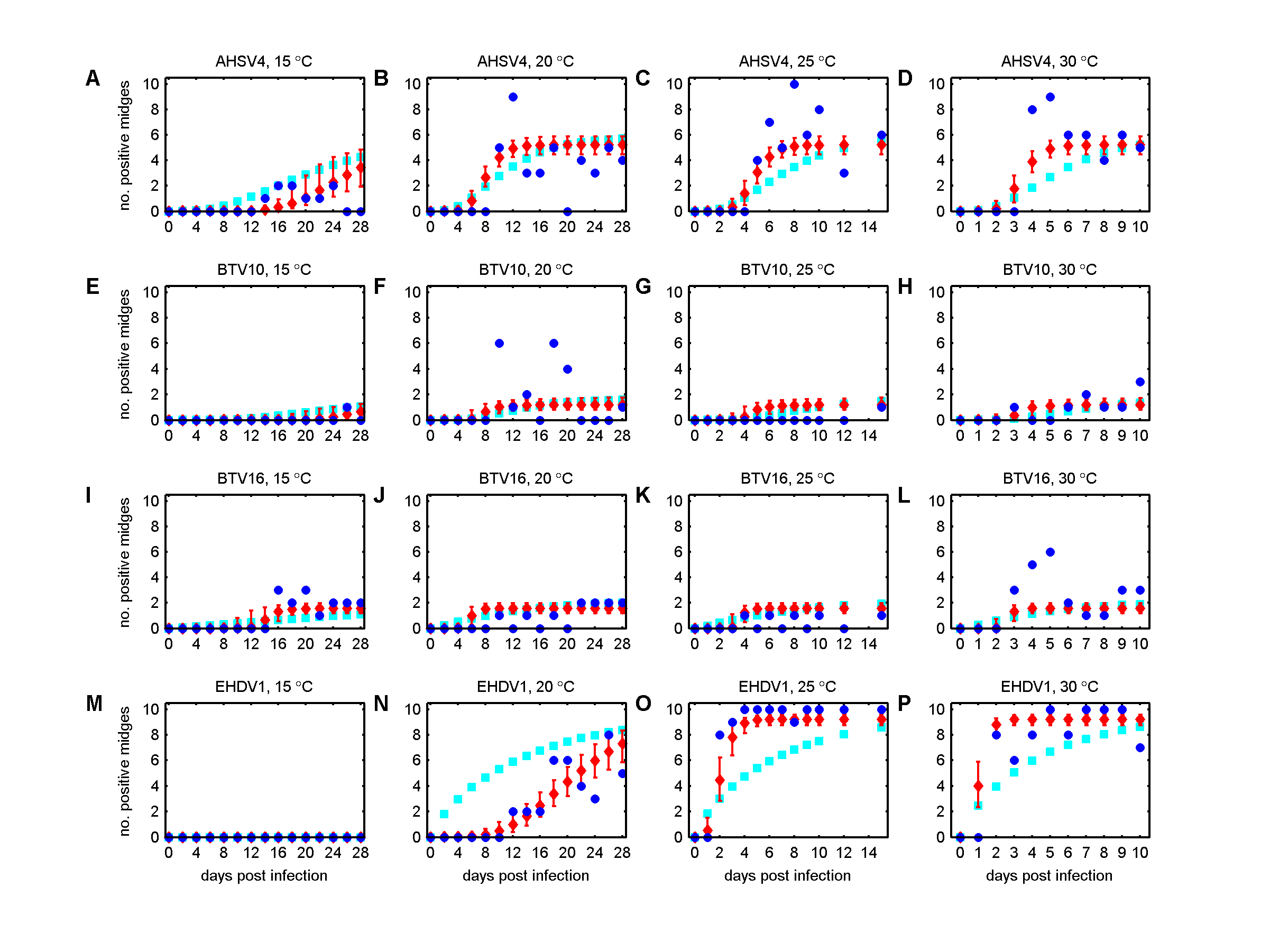

Supplement: Figure S1 — Observed and expected number of Culicoides sonorensis with a fully disseminated infection when reared at different temperatures. Each figure shows the observed (blue circles) and expected (posterior median (red diamonds) and 95% credible interval (error bars)) number of positive C. sonorensis infected with different orbiviruses: African horse sickness virus (AHSV); bluetongue virus (BTV); and epizootic haemorrhagic disease virus (EHDV) (the number indicates serotype). The data were extracted from [15]. The cyan squares show the expected number of positive midges using the estimates for α and T min obtained by [15] with ϕ and k estimated independently by maximum likelihood methods. (TIF) [file pone.0027987.s001.tif]

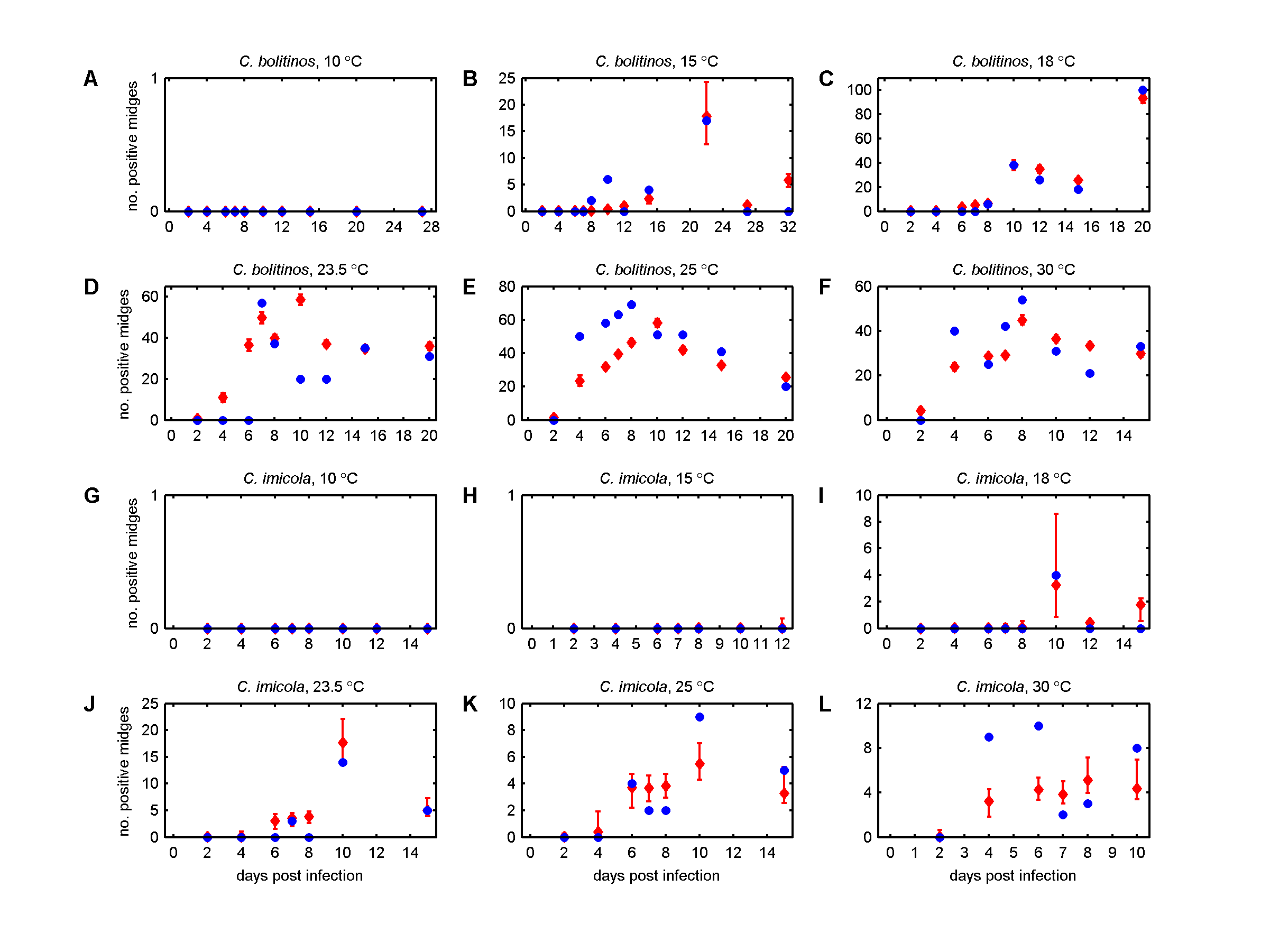

Supplement: Figure S2 — Observed and expected number of Culicoides bolitinos or Culicoides imicola with a fully disseminated infection when reared at different temperatures. Each figure shows the observed (blue circles) and expected (posterior median and 95% credible interval: red diamonds and error bars) number of positive C. bolitinos or C. imicola infected with bluetongue virus serotype 1. The data were extracted from [14]. (TIF) [file pone.0027987.s002.tif]
