# Supplementary material for: Temperature Dependence of the Extrinsic Incubation Period of Orbiviruses in Culicoides Biting Midges
Source: PLoS One. 2011 Nov 18;6(11):e27987. doi: 10.1371/journal.pone.0027987 (PMC3220716; doi:10.1371/journal.pone.0027987)
Supplement: Table S1 — Previous estimates for the temperature dependence of the extrinsic incubation period of different orbiviruses in Culicoides sonorensis. (DOCX) [file pone.0027987.s003.docx]

**Table S1.** Previous estimates for the temperature dependence of the extrinsic incubation period (EIP) of different orbiviruses in *Culicoides sonorensis*.

| orbivirus | virus replication rate (α) | threshold temperature (°C) (*T*_min_) | comments |
| --- | --- | --- | --- |
| AHSV-4* | 0.0085 | 9.7 | - |
| BTV-10* | 0.0069 | 9.2 | - |
| BTV-16* | 0.0113 | 12.6 | - |
| EHDV-1* | 0.0149 | 15.2 | - |
| BTV† | - | 10.4 | based on data for a number of different serotypes; a nonlinear function was fitted to the data |

* Wittmann *et al*. (2002) *Medical and Veterinary Entomology* 16: 147-158.

† Mullens *et al*. (2004) *Veterinaria Italia* 40: 160-166.
